# PD231 A New Way Of Identifying And Selecting Health Technology Assessment Topics: Using Social Media And Citizen Involvement

**DOI:** 10.1017/S0266462324004501

**Published:** 2025-01-07

**Authors:** Yeryeon Jung, Sou Hyun Lee, Hayoung Choi

## Abstract

**Introduction:**

As unfiltered health information overflows through social networking services, people’s health rights are being violated and unnecessary medical expenses are increasing. Citizens and patients participating in the group initiative Public Involvement in the National Evidence-based Healthcare Collaborating Agency (NECA), the sole health technology assessment (HTA) agency in South Korea, began monitoring information on health technology in social media from 2021 as a new way to identify HTA topics.

**Methods:**

Citizens and patients in Public Involvement in NECA were provided with search keywords related to “common diseases on general public” and “diseases with high public interest”. They monitored social media platforms for one month and collected information about health technologies, such as medicines and treatments without scientific evidence on their safety and effectiveness. The target information included subjective information on health technologies based on personal experience; information with unclear sources; information that might cause excessive anxiety and fear; and information with a high risk of side effects. Information was collected from the internet and a checklist was given to the participants to evaluate the suitability of the information.

**Results:**

In the topic selection process, NECA, and the Korean Society of Science Journalists (KSIA) evaluated the priority of HTA topics developed from the collected information. HTA topics were evaluated using five criteria, including the possible harm the information might cause throughout society. As a result, the safety and effectiveness of saw palmetto in patients with prostatic hyperplasia and high-dose intravenous vitamin C in patients with cancer were selected. NECA conducted health technology reassessments on these topics and was able to successfully disseminate the reassessment results in collaboration with the KSIA.

**Conclusions:**

Participation in the HTA process using social media lowered the barriers that laypersons experience with HTA and increased the possibility of bringing HTA topics closer to citizens’ lives. However, as there were difficulties in collecting meaningful information for developing a HTA topic, providing systematic training on health information monitoring remains a challenge.

